# Suppressive Effects of Geoje Raspberry (*Rubus tozawae* Nakai ex J.Y. Yang) on Post-Menopausal Osteoporosis via Its Osteogenic Activity on Osteoblast Differentiation

**DOI:** 10.3390/nu16223856

**Published:** 2024-11-11

**Authors:** Soyeon Hong, Jaeyoung Kwon, Sungmin Song, InWha Park, Da Seul Jung, Erdenebileg Saruul, Chu Won Nho, Hak Cheol Kwon, Gyhye Yoo

**Affiliations:** 1 Smart Farm Research Center, Korean Institute of Science and Technology (KIST), Gangneung 25451, Republic of Korea; hongsso@kist.re.kr (S.H.); na041254@naver.com (D.S.J.); saruul98@gmail.com (E.S.); cwnho@kist.re.kr (C.W.N.); 2 Natural Product Informatics Research Center, Korean Institute of Science and Technology, Gangneung 25451, Republic of Korea; kjy1207@kist.re.kr (J.K.); hmmming95@gmail.com (S.S.); inwha129@naver.com (I.P.); 3 Department of Natural Product Applied Science, University of Science and Technology (UST), Daejeon 34113, Republic of Korea

**Keywords:** osteoporosis, *Rubus tozawae* Nakai ex J.Y. Yang, osteoblast differentiation, osteocalcin, osteogenic activity, ovariectomized mice

## Abstract

Background: Osteoporosis is a metabolic bone disease with a high mortality rate due to non-traumatic fractures. The risk of osteoporosis is increasing globally due to an increasing aging population. Current therapies are limited to delaying disease progression. Recently, the need to discover foods with osteogenic activity for the prevention and treatment of osteoporosis has been emphasized. We focused on bone formation via osteoblast differentiation, considering bone formation and resorption during bone homeostasis. *Rubus tozawae* Nakai ex J. Y. Yang (RL, Geoje raspberry) is a deciduous subshrub that has been traditionally eaten for its fruit. Methods and Results: We identified the third subfraction of n-hexane fraction (RL-Hex-NF3) of RL, an endemic Korean plant with osteogenic activity, which increased bone density in ovariectomized mice, a representative animal model of osteoporosis, via the depletion of female hormones, which resulted from the increase in the osteoblast population. RL-Hex-NF3 induced osteoblast differentiation and the expression of osteogenic markers in MC3T3-E1 pre-osteoblasts. Seven compounds were identified from RL-Hex-NF3 using NMR spectroscopy. Of these, three compounds, namely, 3β-hydroxy-18α,19α-urs-20-en-28-oic acid, betulinic acid, and (1S,6R,7S)-muurola-4,10(14)-diene-15-ol, showed strong osteogenic activity. Conclusions: RL-Hex-NF3 and its compounds suppress bone loss via their osteogenic properties, suggesting that they could be a potent candidate to treat osteoporosis.

## 1. Introduction

Osteoporosis is a skeletal disorder marked by reduced bone density, which significantly increases the likelihood of fractures and correlates with higher mortality from non-traumatic fractures [1,2]. This condition arises from a range of factors, including aging, stress, and genetic predisposition, with estrogen deficiency due to menopause being a primary cause. Studies indicate that approximately 50% of women over 50 years old are affected by osteoporosis during their remaining years [3]. Despite the availability of numerous osteoporosis treatments, ongoing research aims to address the side effects of current therapies, which can include breast cancer, fatty liver disease, and nausea. Consequently, the development of new treatments with fewer adverse effects is essential, spurring research into natural product-based therapies [4,5,6].

Bones are maintained through bone formation by osteoblasts and resorption by osteoclasts. If this harmony of osteoblasts and osteoclasts is broken due to the preceding causes, osteoporosis occurs [7,8,9]. Osteoclasts derived from hematopoietic stem cells are known to play a role in bone resorption. Bone resorption is regulated by osteoclast proliferation and differentiation and the activation of tartrate-resistant acid phosphatase (TRAP). TRAP is an enzyme commonly used as a marker to identify osteoclast activity and bone resorption levels. Various pharmacological osteoporosis treatments have been developed, including bone resorption inhibitors such as bisphosphonates, but those treatments have been reported with severe side effects such as osteonecrosis of the jaw [10,11,12]. In contrast, osteoblasts originating from mesenchymal stem cells have been found to play an important role in bone formation. In the maturation of osteoblasts, three bone morphogenic proteins (BMP), Wnt, and transforming growth factor β (TGF β) signaling are involved. Each signaling pathway is connected by crosstalk and the target gene is expressed through runt-related transcription factor 2 (Runx2). RUNX2 is a transcription factor that expresses target genes, including type 1 collagen 1 (*Col1*), alkaline phosphatase (*Alp*), osterix (*Osx*), osteopontin (*Opn*), and osteocalcin (*Ocn*), that induce osteoblast maturation and bone formation [8,9]. In spite of its important role in bone homeostasis, there are no FDA-approved agents targeting on osteoblast activation.

*Rubus* is a large and diverse genus in the Rosaceae family, with approximately 700 species globally, including raspberries and blackberries [13]. Some *Rubus* species have been used in traditional medicine to treat several diseases including diabetes, infections, and colic. Various phytochemical studies on *Rubus* species have been performed to standardize their chemical composition and identify biologically active compounds [14]. Terpenoids, flavonoids, and phenolic compounds were found to be their main constituents, with diverse biological properties as an anti-inflammatory and antioxidant [15,16]. *Rubus tozawae* Nakai ex J. Y. Yang (RL) is a deciduous subshrub to the southeastern Korean Peninsula. This plant is known as ‘Geoje raspberry’ and it is edible and used for food [17]. To date, no phytochemical or pharmacological studies have been reported on RL. This paper is the first to demonstrate the pharmacological activity of RL. Given the phytochemical diversity and biological properties of *Rubus* species, exploring the biologically active compounds of this unique endemic plant may be worthwhile as it may lead to the discovery of new natural sources with valuable biological activities [18,19]. In this study, we investigated the biological effect of RL on ovariectomized mice, which mimics post-menopausal status including osteoporosis. In addition, we estimated its impact on bone-related signaling using pre-osteoblasts and the bone in ovariectomized mice.

## 2. Materials and Methods

### 2.1. Plant Materials

Whole RL plants were collected in March 2020 from San 48-3, Jisepo-ri, Ilun-myeon, Geoje-si, and Gyeongsangnam-do, South Korea. The collected plant material was authenticated by Jung Hwa Kang (Hantaek Botanical Garden). A voucher specimen (HTS2020-00001) was deposited in the Herbarium of Hantaek Botanical Garden, Yongin, Gyeonggi-do, Republic of Korea. The dried whole plants of *R. tozawae* (7.4 kg) were extracted with 70% EtOH/H_2_O (74 L × 2, 7 d each) at 25 °C. The filtrate was concentrated using a rotary evaporator to obtain 400.0 g of crude extract. A portion of the total extract (350.0 g) was suspended in H_2_O (4 L) and partitioned using *n*-hexane (4 L × 3). The *n*-hexane soluble layer (14.0 g) was subjected to silica gel column chromatography. Seven fractions (NF1–NF7) were obtained through stepwise gradient elution using 500 mL of *n*-hexane/EtOAc (100:0, 20:1, 5:1, 1:1, and 0:100), 500 mL of CH_2_Cl_2_, and 500 mL of acetone. Fraction NF3 (2.3 g) was passed through a Sephadex LH-20 column and eluted with CH_2_Cl_2_/MeOH (1:1) to obtain eight subfractions (NF3-G1 to NF3-G8).

### 2.2. Animal Experiment

All experimental procedures were conducted in accordance with the protocol approved by the Animal Use and Care Committee of the Korean Institute of Science and Technology (KIST-2021-01-003). To construct an osteoporosis-inducing animal model, seven-week-old female C57BL/6J mice were purchased from Central Lab Animal, Inc. (Seoul, Republic of Korea). The mice were maintained under a 12 h light/dark cycle at a constant temperature of 25 ± 2 °C with free access to food and water. Based on previous studies, the sample size for each group was determined to be eight mice. Eight-week-old female C57BL/6J mice (Central Lab Animal Inc., Republic of Korea) were ovariectomized, and a sham operation was performed in the SHAM group (n = 8). OVX mice were randomly divided into four groups: OVX group (n = 8), E+P (0.1 mg/kg β-estradiol and 1 mg/kg progesterone) group (n = 8), RLL (10 mg/kg RL-Hex-NF3) group (n = 8), and RLH (40 mg/kg RL-Hex-NF3) group (n = 8). Each group of mice was housed with four mice per cage, and all procedures that could cause pain were conducted under anesthesia. Surgical procedures were performed under respiratory anesthesia, and dissection was carried out following the administration of anesthetic injections. Blood was collected via cardiac puncture under anesthesia. All animal care followed ethical guidelines. RL-Hex-NF3 concentration was determined based on an in vitro experiment, and the concentration of E+P (0.1 mg/kg β-estradiol and 1 mg/kg progesterone) was established in a previous study [20]. RL-Hex-NF3 and E+P solutions were dissolved in a 0.5% carboxymethyl cellulose solution containing 1% sesame oil and 0.5% DMSO. The SHAM and OVX groups were administered a 0.5% carboxymethyl cellulose solution containing 1% sesame oil and 0.5% DMSO. After 12 weeks of oral administration, blood, uteri, and femurs were collected for further analysis. At the start of the experiment, there were no significant differences in body weight between the groups, and the animals were randomly assigned (Supplementary Table S1).

### 2.3. Primary Cell Isolation

To analyze the osteoblast population in bone, primary bone marrow cells isolated from femurs were cultured. Bone marrow stromal cells (BMSCs) were isolated from mouse femurs as previously described [20]. Cell suspensions were cultured for 24 h in the same culture medium used for MC3T3-E1 cells. Primary cells at passages 2–3 were differentiated using DM treatment for 6 d for further analysis.

### 2.4. Micro-Computed Tomography (Micro-CT)

To analyze the microstructures of mice femurs, Micro-CT was performed according to the method of Bonnet et al. [21]. The distal femurs were scanned using a SkyScan 1172 scanner (Bruker MicroCT Corp., Kontich, Belgium). The scanned bones were analyzed using Nrecon software v1.7.3.2 and CTAn integrated software v.1.17.7.2 (Bruker MicroCT Corp.) to estimate the bone microarchitecture indicators.

### 2.5. Hematoxylin–Eosin Staining (H&E), Immunohistochemistry (IHC), and Serum Parameters

After fixation with 10% *v*/*v* formalin, the paraffin-embedded femoral bones were sectioned at 5 μm, and stained with H&E, COL1, and TRAP antibody (Abcam, UK). COL1- and TRAP-positive areas were quantified using ImageJ software v.2.16.0 (National Institute of Health, Bethesda, MD, USA). Serum osteocalcin levels were measured using a Gla-type osteocalcin (Gla-OC) EIA Kit.

### 2.6. Cell Culture

MC3T3-E1 mouse pre-osteoblast cells (ATCC, Manassas, VA, USA) were cultured in minimum essential medium Eagle-Alpha modification (α-MEM) (Gibco, Gaithersburg, MD, USA) containing fetal bovine serum (Gibco) and 1% penicillin–streptomycin (P/S, Gibco) in a 5% CO_2_ incubator at 37 °C in an atmosphere of 5% CO_2_. After reaching 70% confluence, the cells were trypsinized and passaged.

### 2.7. ALP Activity

Osteoblast differentiation experiments were performed with a differentiation medium (DM) that contained 50 μg/mL l-ascorbic acid and 10 mM β-glycerophosphate (St. Louis, MO, USA). The extract, fractions, or compounds were treated MC3T3-E1 cells with DM. After 6 d in DM, the cells were lysed with 0.1% Triton-X-100 lysis buffer and alkaline phosphatase activity was measured at 405 nm using a SensoLyte pNPP Alkaline Phosphatase Assay Kit (AnaSpec, Fremont, CA, USA).

### 2.8. Western Blotting and RT-PCR

For protein and mRNA expression analyses, MC3T3-E1 cells were treated with DM-containing fractions and specific compounds for 6 days. Protein expression was analyzed using Western blotting, as previously described [20]. Antibodies targeting β-actin, RUNX2, OPN, OSX, BMP2/4, TGFβ, and COL1 were obtained from Santa Cruz Biotechnology (Dallas, TX, USA), while antibodies against β-catenin, p-Smad1/5, and p-Smad2/3 were sourced from Cell Signaling Technology (Danvers, MA, USA). β-actin served as an internal control to ensure consistent protein loading across samples. Secondary anti-mouse and anti-rabbit antibodies were also utilized in the analysis. For mRNA expression analysis, qRT-PCR was conducted following the method outlined previously [20]. Specific PCR primers were used to measure mRNA levels, which were normalized to GAPDH expression as a reference gene (Table 1).

### 2.9. Chemical Analysis and Isolation of Compounds

Nuclear magnetic resonance (NMR) spectroscopy was performed using a Bruker 500 MHz NMR system. Mass spectral data were acquired using an Agilent 1200 series instrument equipped with a 6120 quadrupole LC-MS instrument. Thin-layer chromatography (TLC) was conducted on Merck aluminum sheets precoated with silica gel 60 F254. Size-exclusion chromatography was performed with Sephadex LH-20 (18−111 μm). Preparative HPLC was performed on a YMC LC-Forte/R system with an evaporative light scattering detector using a YMC SIL column (250 × 10 mm i.d., 5 μm). Column chromatography was performed using a Pasteur pipette (150 × 6.3 mm) filled with silica resin.

Fractions NF3-G2, NF3-G3, NF3-G6, NF3-G7, and NF-G8 were sequentially subjected to semi-preparative, normal-phase HPLC (YMC SIL column, 250 × 10 mm; 5.0 mL/min; *n*-hexane/EtOAc 6:1) to obtain the respective compounds and subfractions. Fraction NF3-G2 was separated to yield pheophorbide A (6; 11.9 mg) and other subfractions (NF3-G2-5, NF3-G2-18, and NF3-G2-26). Fraction NF3-G3 was separated to obtain a triglyceride derivative (7; 20.8 mg) and further subfractions (NF3-G3-4, NF3-G3-7, and NF3-G3-14). Fraction NF3-G6 was separated to yield phytol (4; 39.9 mg) and β-sitosterol (5; 106.0 mg). Fraction NF3-G7 was separated to obtain 3*β*-hydroxy-18*α*,19*α*-urs-20-en-28-oic acid (1; 0.5 mg), betulinic acid (2; 0.3 mg), and further subfractions (NF3-G7-10c and NF3-G7-10d). Fraction NF3-G8 (18.1 mg) was separated using the same conditions as NF3-G2 and then further purified using a silica gel (6.3 × 150 mm) column with CH_2_Cl_2_ to produce (*1S*,*6R*,*7S*)-muurola-4,10(14)-diene-15-ol (3; 1.6 mg) and further subfractions (NF3-G8-8 and NF3-G8-9). Detailed NMR data for the active compounds are described below (Supplementary Figure S1).

*3β-hydroxy-18α,19α-urs-20-en-28-oic acid*. ^1^H NMR (Pyridine-*d*_5_, 500 MHz) *δ* 5.76 (1H, d, *J* = 5.5 Hz, OH-3), 5.51 (1H, d, *J* = 7.0 Hz, H-21), 3.50 (1H, m, H-3), 2.89 (1H, m, H-13), 2.67 (1H, dd, *J* = 15.5, 7.0 Hz, H_2_-22), 2.48 (1H, t, *J* = 6.6 Hz, H-19), 2.35 (1H, dt, *J* = 13.1, 3.5 Hz, H_2_-12), 2.04 (1H, m, H_2_-22), 1.95–1.70 (8H, m, H_2_-2, H_2_-15, H_2_-16, H_2_-1), 1.75 (3H, s, H_3_-30), 1.66–1.50 (4H, m, H_2_-6, H-5, H_2_-12), 1.41 (2H, m, H_2_-7), 1.37–1.32 (4H, m, H_2_-11, H-9, H-18), 1.26 (3H, s, H_3_-24), 1.14 (3H, d, *J* = 6.6 Hz, H_3_-29), 1.09 (3H, s, H_3_-26), 1.07 (3H, s, H_3_-27), 1.05 (3H, s, H_3_-23), 0.89 (3H, s, H_3_-25); ESIMS *m*/*z* 457 [M + H]^+^.

*Betulinic acid*. ^1^H NMR (CDCl_3_, 500 MHz) *δ* 4.74 (1H, s, H-29), 4.61 (1H, s, H-29), 3.19 (1H, dd, *J* = 10.8, 4.8 Hz, H-3), 2.99 (1H, td, *J* = 10.8, 5.0 Hz, H-19), 2.26 (1H, m, H_2_-16), 2.21 (1H, m, H-13), 2.03–1.92 (2H, m, H_2_-22, H_2_-21), 1.72 (1H, m, H_2_-12), 1.69 (3H, s, H_3_-30), 1.68–1.64 (2H, m, H-18, H_2_-2), 0.97 (3H, s, H_3_-27), 0.96 (3H, s, H_3_-24), 0.94 (3H, s, H_3_-26), 0.82 (3H, s, H_3_-25), 0.75 (3H, s, H_3_-23); ESIMS *m*/*z* 457 [M + H]^+^.

*(1S,6R,7S)-Muurola-4,10(14)-diene-15-ol*. [*α*]^20^_D_ +9.6 (*c* 0.04, MeOH); ^1^H NMR (CDCl3, 500 MHz): *δ* 5.85 (1H, br d, *J* = 5.1 Hz, H-5), 4.68 (1H, br s, H_2_-14), 4.62 (1H, br s, H_2_-14), 4.03 (2H, br s, H_2_-15), 2.41 (1H, dt, *J* = 13.4, 4.1 Hz, H-1), 2.20 (1H, m, H_2_-9), 2.13 (1H, m, H_2_-9), 2.11 (2H, m, H_2_-3), 2.03 (1H, m, H-6), 1.99 (1H, m, H-11), 1.94 (1H, m, H_2_-2), 1.70 (1H, m, H_2_-8), 1.51 (1H, m, H_2_-2), 1.44 (1H, dt, *J* = 11.5, 3.4 Hz, H-7), 1.04 (1H, m, H_2_-8), 0.92 (3H, d, *J* = 7.0 Hz, H_3_-12), 0.81 (3H, d, *J* = 7.0 Hz, H_3_-13); ESIMS *m*/*z* 221 [M + H]^+^.

### 2.10. Statistical Analysis

Data are presented as mean ± standard error of mean (SEM) and were analyzed using GraphPad Prism 9.0, followed by Duncan’s multiple range test. Differences were considered statistically significant at *p* < 0.05.

## 3. Results

### 3.1. Oral Administration of RL-Hex-NF3 Prevents Osteoporotic Bone Loss in OVX Mice via Osteoblast Differentiation

First, we prepared the RL extract and its fractions, *n*-hexane and *n*-butanol, and estimated their effect on the activity of ALP, an early marker of osteoblast differentiation. EtOH extract and fractions of RL were treated in MC3T3E1 cells for ALP activity, and the activity was found to be elevated most by the treatment of RL *n*-hexane fraction (RL-Hex) (Supplementary Figure S2A). Additional bioassay-guided fractionation showed that fraction 3 of RL-Hex (RL-Hex-NF3) significantly increased ALP activity compared to the other fractions, similar to that of RL-Hex (Supplementary Figure S2B). Based on these data, RL-Hex-NF3 was selected for subsequent experiments.

**Figure 1:**
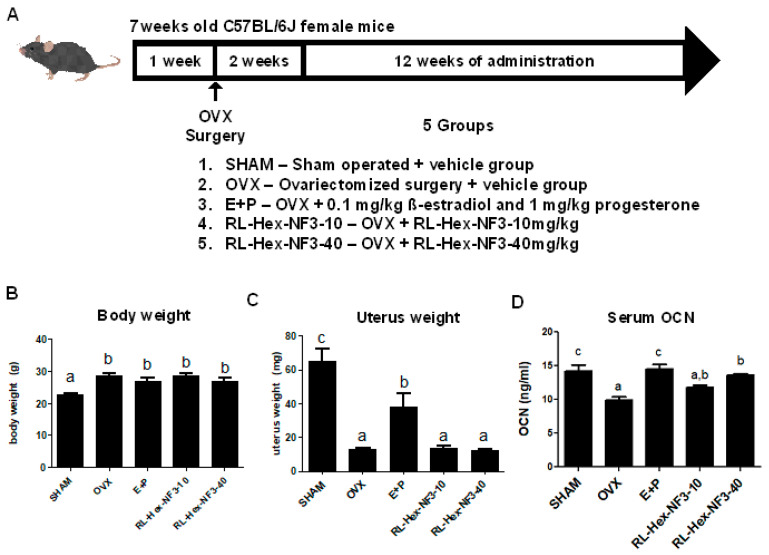
Basic data of osteoporosis mouse experiment. (**A**) Design of the animal experiment. (**B**) Body weight and (**C**) uterus weight of OVX mice. (**D**) Serum osteocalcin levels measured using ELISA. Different letters represent significant differences (*p* < 0.05).

To evaluate the impact of RL-Hex-NF3 on the bone, RL-Hex-NF3 was orally administered to OVX mice, a model of post-menopausal osteoporosis (Figure 1A). The administration of RL-Hex-NF3 did not significantly affect body weight and induced no noticeable changes in uterine weight (Figure 1B,C). Serum OCN levels, which decreased following OVX treatment, were increased following RL-Hex-NF3 administration (Figure 1D).

**Figure 2:**
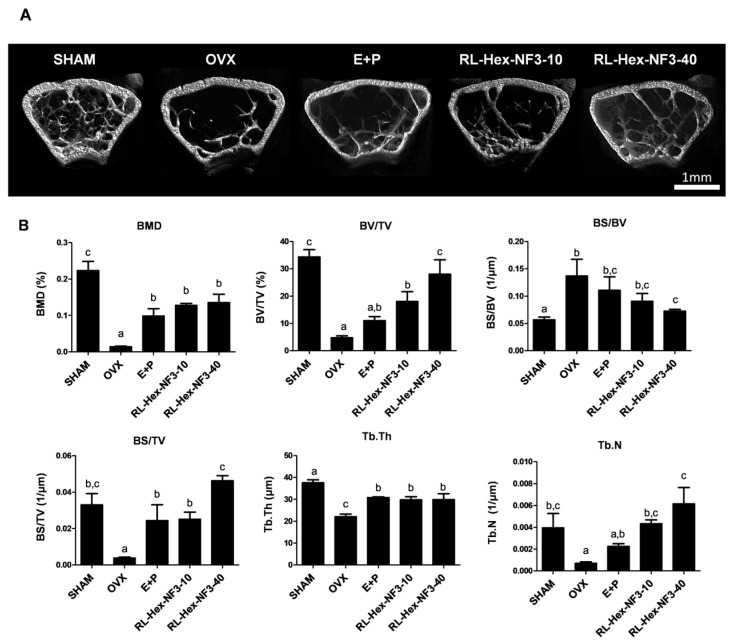
Effects of RL-Hex-NF3 on the bone status in OVX mice. (**A**) Micro-CT images and (**B**) bone microarchitecture indicators of the distal femoral region of mice from each group. Bone mineral density (BMD), bone volume/total volume (BV/TV), bone surface area/bone volume (BS/BV), bone surface area/total volume (BS/TV), trabecular thickness (Tb.Th), and trabecular plate number (Tb.N) were determined using computed tomography. Different letters represent significant differences (*p* < 0.05).

The micro-CT analysis of the femurs revealed that OVX mice exhibited lower bone mineral density (BMD) (Figure 2A,B). The oral administration of RL-Hex-NF3 substantially restored bone density. Key bone turnover markers, such as bone volume/total volume (BV/TV), bone surface area/total volume (BS/TV), and trabecular thickness (Tb.Th), were decreased by OVX and subsequently recovered following RL-Hex-NF3 treatment. Conversely, the bone surface area/bone volume (BS/BV), another marker of bone turnover, increased due to OVX and decreased following RL-Hex-NF3 administration (Figure 2B).

**Figure 3:**
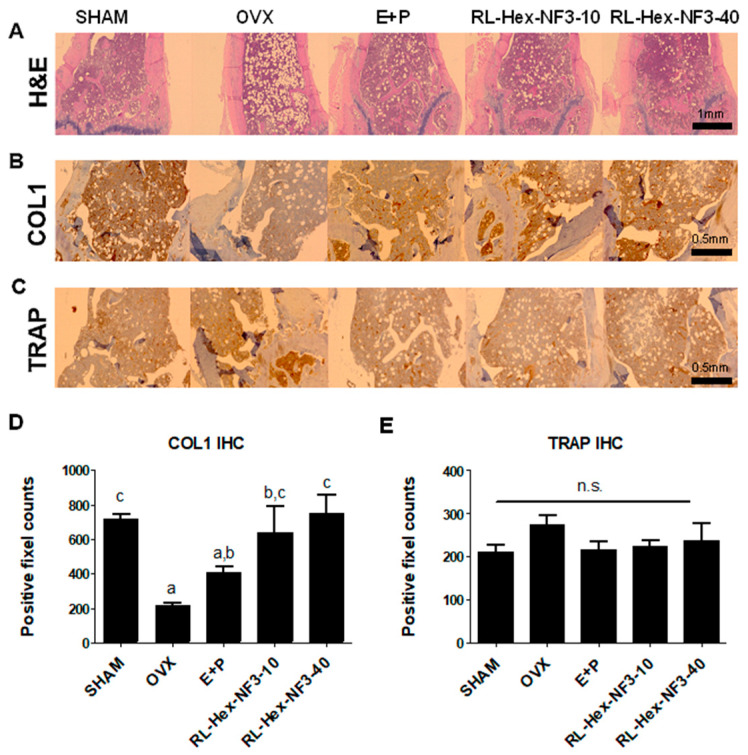
Histological observation of the distal femoral region of bones. (**A**) H&E staining, (**B**,**D**) type 1 collagen staining, and (**C**,**E**) TRAP staining of the distal femoral region of mice from each group. Different letters represent significant differences (*p* < 0.05); n.s. means not significant.

The hematoxylin and eosin (H&E) staining of the femurs confirmed that OVX surgery increased the empty spaces within the femurs, while hormone and RL-Hex-NF3 treatments significantly reduced these spaces (Figure 3A). Immunohistochemistry (IHC) indicated that the collagen type 1 (COL1) content within the femurs was significantly reduced by OVX surgery and significantly increased by RL-Hex-NF3 treatment (Figure 3B,D). Regarding tartrate-resistant acid phosphatase (TRAP), an indicator of osteoclast differentiation, no statistically significant difference was observed after RL-Hex-NF3 administration (Figure 3C,E).

**Figure 4:**
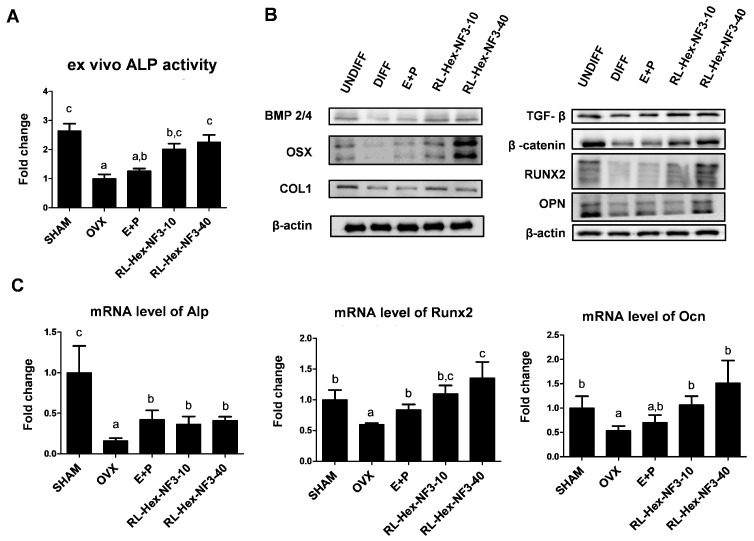
Effects of RL-Hex-NF3 on the osteoblast population of bone marrow. (**A**) ALP activity, (**B**) protein expression, and (**C**) mRNA expression levels of osteoblastic markers in primary bone marrow cells. Different letters represent significant differences (*p* < 0.05).

The ALP activity decreased after OVX and increased after RL-Hex-NF3 treatment, indicating the presence of more osteoblasts (Figure 4A). Major osteoblast stimulators, such as β-catenin, TFG β, and BMP2/4, were decreased by OVX, but increased in the OVX mice following RL-Hex-NF3 treatment. In addition, the osteoblastic markers, OPN, OSX, RUNX2, and COL1, were decreased in OVX mice, but recovered after RL-Hex-NF3 treatment (Figure 4B). Similarly to protein expression, the mRNA expression of Alp, Runx2, and Ocn was decreased by OVX surgery and increased in RL-Hex-NF3-treated mice (Figure 4C).

### 3.2. RL Hexane Fraction Induces Osteoblastic Differentiation

**Figure 5:**
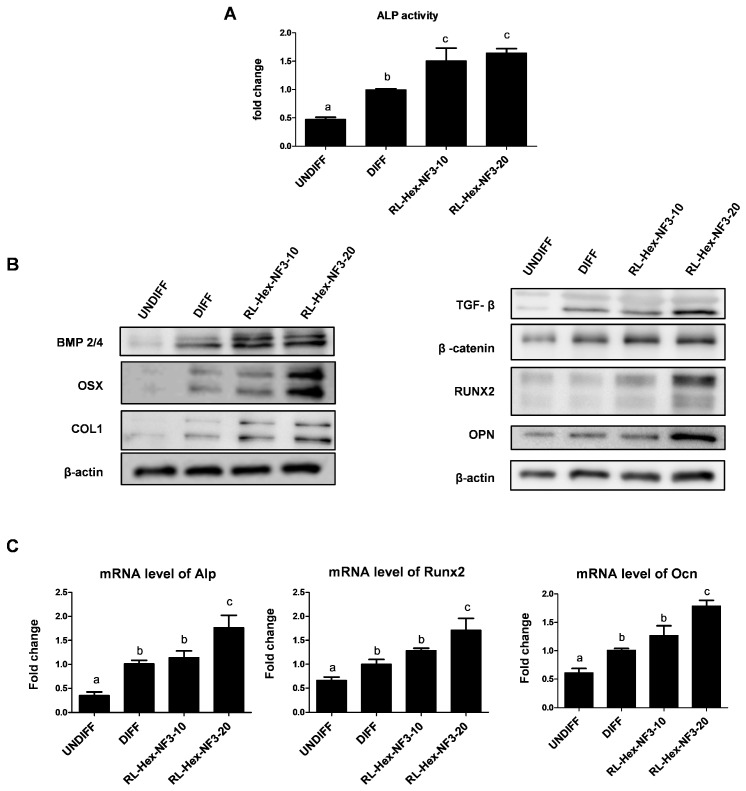
Effects of the RL extract, fractions, and new subfractions (NF) on osteoblastic differentiation in vitro. (**A**) ALP activity of RL-Hex-NF3. (**B**) Protein and (**C**) mRNA expression levels of osteoblastic markers as determined using Western blotting and qRT-PCR. UNDIFF indicates MC3T3E1 cells in cultured media; DIFF indicates MC3T3E1 cells in differentiation media (DM); 10 and 20 indicate MC3T3E1 cells in DM with 10 μg/mL and 20 μg/mL of RL-Hex-NF3, respectively. Different letters represent significant differences (*p* < 0.05).

Because RL-Hex-NF3 has suppressive effect on bone loss via the increase in the osteoblast population, we evaluated its osteogenic activity in osteoblast differentiation. To clarify the mechanism of RL-Hex-NF3 on osteoblasts, a differentiation experiment using MC3T3-E1 pre-osteoblasts was performed. RL-Hex-NF3 was shown to significantly increase ALP activity at 10–20 µg/mL (Figure 5A). RL-Hex-NF3 increased protein expression levels of β-catenin and BMP2/4 but not TGF-β, which are major signaling molecules of osteoblast differentiation (Figure 5B). Transcription factors modulating osteoblast differentiation, namely, p-smad 2/3, p-smad 1/5, and RUNX2, were also increased, and the markers of osteoblast differentiation OPN and COL1, were increased following treatment with 10 µg/mL and 20 µg/mL of RL-Hex-NF3 in MC3T3-E1 cells. In the mRNA expression analysis, Alp, Ocn, and Runx2 were found to be elevated by the 10 µg/mL and 20 µg/mL treatment of RL-Hex-NF3 (Figure 5C). This has been proven that these in vitro results were similar to the animal experiment results.

### 3.3. Compounds ***1***–***3*** from RL-Hex-NF3 Increase Osteoblast Differentiation

**Figure 6:**
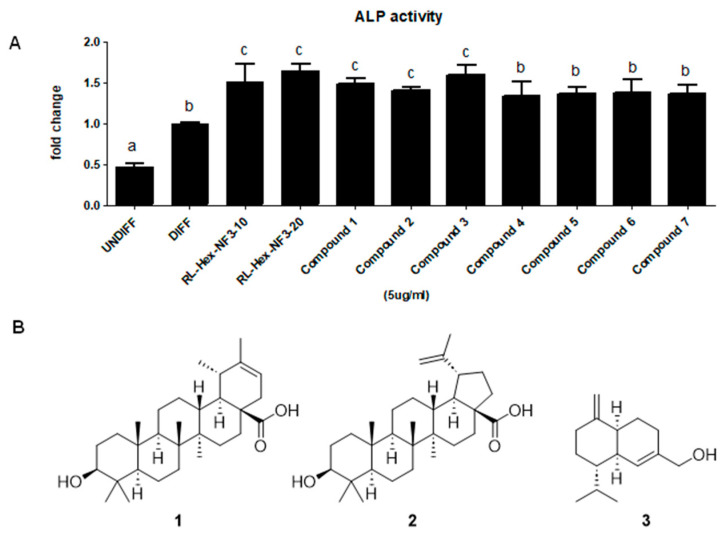
(**A**) ALP activity of compounds from RL-Hex-NF3. Different letters represent significant differences (*p* < 0.05). (**B**) Chemical structures of the compounds from RL-Hex-NF3: (**1**) 3β-hydroxy-18α,19α-urs-20-en-28-oic acid, (**2**) betulinic acid, and (**3**) (1S,6R,7S)-muurola-4,10(14)-diene-15-ol.

To identify the active molecules of RL-Hex-NF3 impacting osteoblast differentiation, we obtained seven compounds from RL-Hex-NF3 using several different chromatographic techniques. All compounds were re-tested using the ALP screening system, and three compounds were found to be biologically active (Figure 6A). Of these, compounds **1**–**3** showed the best induction of ALP activity but were less effective than RL-Hex-NF3. The spectroscopic and spectrometric analysis using NMR and HPLC-MS resulted in the structural characterization of three compounds as 3β-hydroxy-18α,19α-urs-20-en-28-oic acid (compound **1**), betulinic acid (compound **2**), and (*1S*,*6R*,*7S*)-muurola-4,10(14)-diene-15-ol (compound **3**) (Figure 6B) [22,23,24].

## 4. Discussion

Currently, with the exception of hormone therapy, most therapies for osteoporosis focus on preventing bone resorption [25]. However, these treatments have two drawbacks: severe side effects, such as esophageal cancer, hypocalcemia, ocular inflammation, osteonecrosis of the jaw, and nephrotoxicity [26], and treatment failure to recover a bone status similar to that of healthy bone. Our findings suggest that RL has an anti-osteoporotic effect via osteogenic activity on osteoblasts, but not on osteoclasts. Our data showed that RL-Hex-NF3 increased osteogenic markers in vitro, increasing BMP 2/4 and Wnt signaling, osteogenic transcription factors, and genes involved in calcium attachment and hydroxyapatite formation. In addition, RL-Hex-NF3 treatment increased bone parameters, including bone mineral density and osteoblast population, in mouse femurs in vivo, resulting in higher bone density. Furthermore, we confirmed through additional experiments that these effects originated from three active compounds present in RL-Hex-NF3.

Osteoblasts are generally activated through three signaling pathways: TGFβ, BMP 2/4, and Wnt. TGFβ signaling is involved in many cellular processes in both adult organisms and developing embryos, including cell growth, cell differentiation, cell migration, apoptosis, cell homeostasis, and other cellular functions [27]. Although BMPs were initially discovered for their ability to induce bone formation, they are now known to play important roles in embryonic development and adult tissue homeostasis [28]. Wnt signaling plays a role in cell fate det5ermination, cell migration, cell polarity, neural patterning, and organogenesis during embryonic development [29]. These three pathways play a role in bone formation during development by regulating the expression of Runx2 and Osx. We examined all three pathways following RL-Hex-NF3 treatment and found that BMP 2/4, and Wnt were activated by RL-Hex-NF3. This confirms the view in the existing literature that these signals do not work independently but interact with each other through crosstalk [30]. We confirmed that the activation of these signaling pathways led to an increase in RUNX2 and OSX and the subsequent elevation of COL1 in vitro and in vivo. Two osteogenic transcription factors, RUNX2 and OSX, have been shown to interact and modulate the expression of target genes such as *Col1*. Collagen is the most abundant protein in bones and is known to play a role in stably anchoring minerals to bones [31]. Existing research has demonstrated that as the quantity and quality of collagen increases, bone strength improves, and our results confirm those of previous studies.

Osteocalcin (OCN), the predominant protein in bone, serves as a specific marker for osteoblast differentiation and is exclusively secreted by osteoblasts. It plays a vital role in bone metabolism by facilitating calcium binding, thus maintaining and regenerating bone tissue. Furthermore, OCN acts as a hormone released into the bloodstream, influencing muscle function and blood sugar regulation. In the early stages of menopause, the destruction of osteoblasts leads to a temporary increase in serum OCN levels, which subsequently decline as post-menopausal osteoporosis progresses [32,33]. Our research demonstrates that OCN expression rises during the differentiation of osteoblasts, suggesting that this process allows for the maturation of osteoblasts and their ability to incorporate calcium into the bone matrix. In vivo studies revealed that OVX mice exhibited a decrease in serum OCN levels, confirming the onset of osteoporosis. However, following the oral administration of RL-Hex-NF3, there was a notable increase in OCN levels in both serum and bone marrow cells, which was further validated by mRNA expression analyses. These findings suggest that RL-Hex-NF3 promotes the maturation of osteoblasts and effectively prevents the progression of osteoporosis by inhibiting osteoblast destruction within the bone. However, the level of TRAP in the femurs of mice was not changed by RL-Hex-NF3, indicating that the effect of RL-Hex-NF3 was mediated by the differentiation and activation of osteoblasts rather than osteoclasts. Numerous studies have shown that ovariectomy (OVX) induces changes in TRAP levels. In our study, the absence of such changes can be attributed to two possibilities: first, that the timing of the dissection did not allow for the sufficient induction of TRAP changes, and second, that RL primarily activates osteoblasts, which may have resulted in a lower expression of TRAP levels.

Seven compounds from RL-Hex-NF3 were isolated to determine which compounds within RL affected osteoblasts [7,8,9,34]. Of these compounds, 3β-hydroxy-18α,19α-urs-20-en-28-oic acid (compound **1**), betulinic acid (compound **2**), and (1S,6R,7S)-muurola-4,10(14)-diene-15-ol (compound **3**) had the highest ALP activity, indicating that they are major contributors to the osteogenic activity of RL-Hex-NF3. Betulinic acid is known to activate osteoblast differentiation through the BMP2/4 signaling pathway and inhibit osteoclastogenesis [22]. Although these three compounds showed significant activity, RL-Hex-NF3 was the most effective, suggesting that the therapeutic effect of RL on osteoporosis may be attributed to the synergistic effects of multiple compounds. While we could not determine which specific signaling pathways are activated by each compound in relation to osteoblast activation, we hypothesize that the synergistic effect of these three compounds may enhance osteoblast differentiation. However, further studies are needed to confirm these interactions. Our findings suggest that the oral administration of RL-Hex-NF3 may be a better option to prevent osteoporosis through osteoblast differentiation and demonstrate that 3β-hydroxy-18α,19α-urs-20-en-28-oic acid and (1S,6R,7S)-muurola-4,10(14)-diene-15-ol play a critical role in the suppressive effect of RL-Hex-NF3 via their osteogenic activity.

## 5. Conclusions

We found that the administration of RL-Hex-NF3 suppressed bone loss by inducing osteoblast differentiation. This observed osteo-protective action is likely due to the synergistic effects of three prominent compounds, (**1**) 3β-hydroxy-18α,19α-urs-20-en-28-oic acid, (**2**) betulinic acid, and (**3**) (1S,6R,7S)-muurola-4,10(14)-diene-15-ol. Our findings suggest that these effects are mediated via the upregulation of Runx2, a key transcription factor in osteoblast differentiation, along with the increased expression of its target genes, such as OSX and collagen. Collectively, our results indicate that the hexane fraction of RL holds promise as a novel preventive and therapeutic agent for osteoporosis.

## Figures and Tables

**Table 1:** Sequences of PCR primers.

Gene	Forward (5′-3′)	Reverse (5′-3′)
Runx2	TCCACAAGGACAGAGTCAGATTAC	TGGCTCAGATAGGAGGGGTA
ALP	GATCATTCCCACGTTTTCAC	TGCGGGCTTGTGGGACCTGC
Ocn	AGACTCCGGCGCTACCTT	CTCGTCACAAGCAGGGTTAAG
Gapdh	AAGAGGGATGCTGCCCTTAC	CCATTTTGTCTACGGGACGA

## Data Availability

The original contributions presented in the study are included in the article, and further inquiries can be directed to the corresponding author.

## Supplementary Materials

The following supporting information can be downloaded at: https://www.mdpi.com/article/10.3390/nu16223856/s1, Table S1: Body weight of mice. Data are presented as mean ± standard error of mean (SEM). Supplementary Figure S1. 1H NMR data of active compounds and Supplementary Figure S2. Basic physiological data in vivo.
