# Supplementary material for: Suppressive Effects of Geoje Raspberry (Rubus tozawae Nakai ex J.Y. Yang) on Post-Menopausal Osteoporosis via Its Osteogenic Activity on Osteoblast Differentiation
Source: Nutrients. 2024 Nov 11;16(22):3856. doi: 10.3390/nu16223856 (PMC11597101; doi:10.3390/nu16223856)
Supplement: Supplementary file 1 [file nutrients-16-03856-s001.zip › Supplementary Table S1. Body weight of mice.pdf]

Groups	Week 0 (g)	Week 14 (g)
SHAM	17.09 ± 0.588	22.29 ± 0.440 [a]
OVX	17.19 ± 0.350	28.10 ± 0.750 [b]
E+P	17.22 ± 0.342	27.16 ± 0.947 [b]
RL-Hex-NF3-10	17.21 ± 0.322	28.30 ± 0.720 [b]
RL-Hex-NF3-40	17.05 ± 0.262	25.95 ± 1.273 [b]

Supplymentary Table S1. Body weight of mice. Data are presented as mean ± standard error of mean (SEM).
