# Supplementary figures and images for: Suppressive Effects of Geoje Raspberry (Rubus tozawae Nakai ex J.Y. Yang) on Post-Menopausal Osteoporosis via Its Osteogenic Activity on Osteoblast Differentiation
Source: Nutrients. 2024 Nov 11;16(22):3856. doi: 10.3390/nu16223856 (PMC11597101; doi:10.3390/nu16223856)

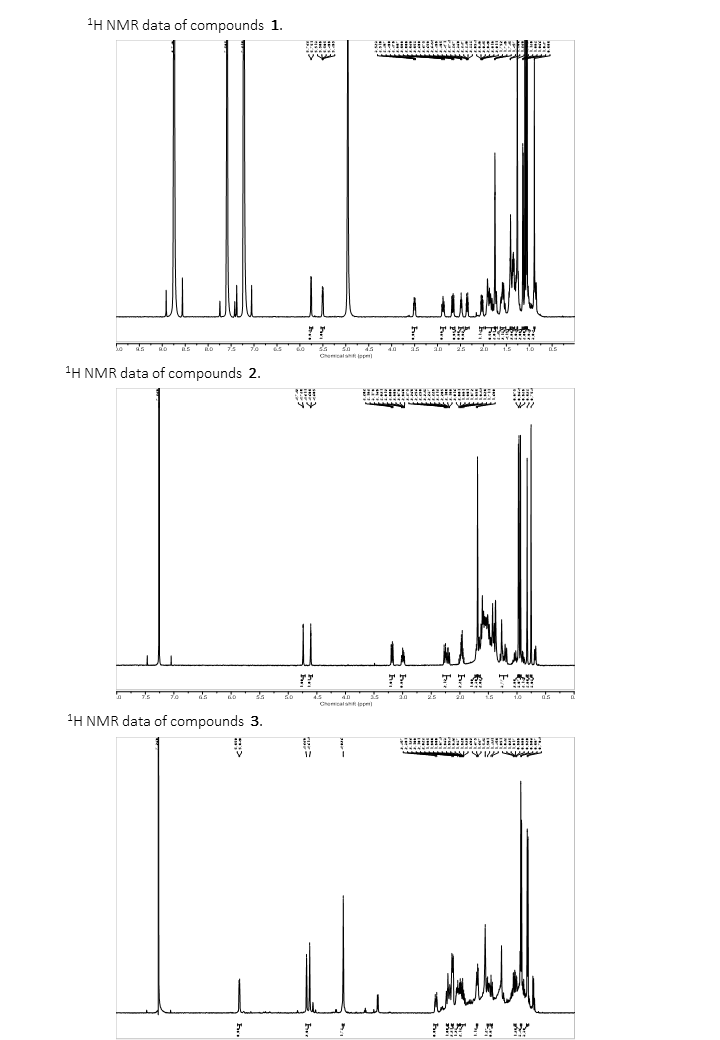

Supplement: Supplementary file 1 [file nutrients-16-03856-s001.zip › supplementary figure S1.tif]

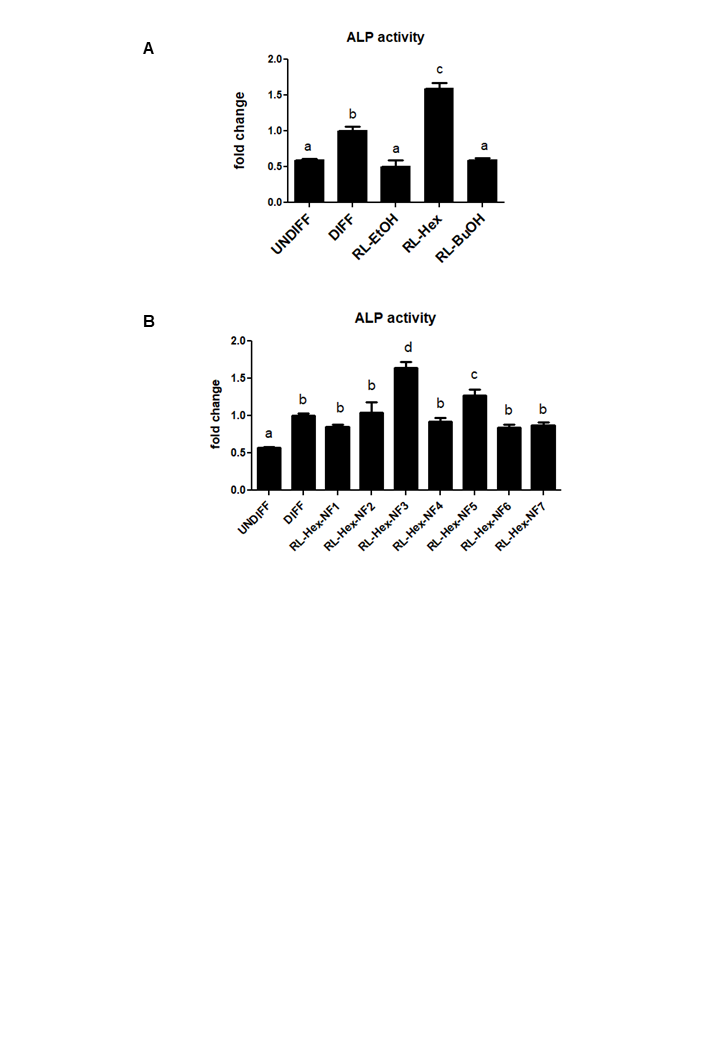

Supplement: Supplementary file 1 [file nutrients-16-03856-s001.zip › supplementary figure S2.tif]
